# Echolaser Focal Treatment for Prostate Cancer Guided by Fiducial Marker Placement

**DOI:** 10.3390/cancers17101707

**Published:** 2025-05-20

**Authors:** Timoleon Granitsas, Ioannis Anastassakis, Stamatios Brempos, Kyriakos Brempos

**Affiliations:** 1 Athens Medical Center, Psychico Clinic, 11526 Athens, Greece; timoleongranitsas@yahoo.gr (T.G.); ianastassakis@gmail.com (I.A.); 2 Faculty of Farmacy and Medicine, Sapienza University of Rome, 00185 Roma, Italy; brempos.1923833@studenti.uniroma1.it

**Keywords:** prostate cancer, focal treatment, EchoLaser, fiducial markers

## Abstract

**Background**: Focal therapy has emerged as a viable alternative to radical prostate cancer treatment, offering oncologic control while minimizing morbidity. EchoLaser focal laser ablation (FLA) is a minimally invasive technique that utilizes high-precision laser energy for tumor destruction. This study evaluated the oncologic outcomes, procedural efficiency, and safety of EchoLaser focal therapy, comparing fiducial-assisted (FM+) and non-fiducial (FM−) approaches. **Methods**: A retrospective cohort study was conducted at Athens Medical Center, Greece, including 50 patients with localized prostate cancer treated with EchoLaser therapy. Patients were categorized into FM+ (n = 31) and FM− (n = 19) groups. Oncologic control (MRI and PSA levels at six months), procedural efficiency (operative time), and safety (adverse events) were assessed. **Results**: At six months, 80% of patients (n = 40) had no residual disease on MRI, while 20% (n = 10) showed persistent or recurrent tumor activity. PSA levels declined from 10.26 ± 14.99 ng/mL to 2.70 ± 2.67 ng/mL, reflecting a 74% median reduction. Procedure time was shorter in FM+ patients (33.48 ± 2.41 min vs. 45.79 ± 2.92 min, *p* < 0.01). Adverse events occurred only in the FM− group, including one case of urinary retention. **Conclusions**: FLA with EchoLaser using fiducial marker enhances procedural efficiency and could have a positive impact on oncologic control. These findings suggest that fiducial markers should be integrated into focal therapy protocols. Longer follow-up studies are needed to confirm the long-term outcomes.

## Simple Summary

Prostate cancer is one of the most common cancers in men, and treatment options often involve surgery or radiation, which can lead to side effects such as urinary incontinence and erectile dysfunction. Focal therapy is a newer approach that targets only the cancerous part of the prostate, preserving healthy tissue and reducing side effects. This study examined the use of EchoLaser Fusion guided laser ablation, a minimally invasive treatment that destroys cancer cells with heat, comparing two methods: one with fiducial markers, which help doctors precisely target the tumor, and one without. The results showed that patients who received fiducial markers had better treatment success, fewer recurrences, and shorter procedure times. These findings suggest that fiducial-assisted laser therapy may improve the accuracy and effectiveness of focal prostate cancer treatment, helping to refine non-surgical treatment options and offering men a safer, more precise alternative to traditional therapies.

## 1. Introduction

Prostate cancer (PCa) remains one of the most prevalent malignancies affecting the male population worldwide [1,2,3]. According to global cancer statistics, PCa is the second most frequently diagnosed cancer among men and the fifth leading cause of cancer-related mortality [4,5,6]. The disease exhibits a wide spectrum of clinical behavior, ranging from indolent, localized tumors with minimal progression to aggressive, high-grade malignancies with significant metastatic potential [7,8,9,10]. This heterogeneity underscores the need for a personalized treatment approach that balances oncologic control with quality-of-life preservation.

For patients diagnosed with low- or intermediate-risk PCa, active surveillance is often recommended as the primary management strategy to avoid overtreatment and its associated morbidities [11,12,13]. Active surveillance involves regular monitoring of prostate-specific antigen (PSA) levels, digital rectal exams (DRE), multiparametric magnetic resonance imaging (mpMRI), and periodic biopsies to detect any signs of disease progression [14,15]. However, studies indicate that more than 50% of patients initially placed under active surveillance ultimately require definitive treatment due to disease progression, rising PSA levels, or increased Gleason scores on subsequent biopsies [16,17,18].

Traditionally, localized prostate cancer is managed with radical prostatectomy or external beam radiotherapy (EBRT), both of which have demonstrated high efficacy in achieving long-term disease control [19,20]. However, despite their oncologic benefits, these radical therapies are associated with significant treatment-related morbidities, particularly urinary incontinence, erectile dysfunction, and bowel dysfunction [19,21,22]. Studies have reported that post-prostatectomy urinary incontinence rates range from 3% to 20%, while erectile dysfunction affects up to 79% of patients [23,24,25,26]. Similarly, EBRT is associated with radiation-induced proctitis, bladder irritation, and potential secondary malignancies [27,28,29].

Given these concerns, there has been a paradigm shift in the management of localized PCa, focusing on less invasive and organ-preserving techniques that aim to reduce morbidity while maintaining oncologic efficacy. The growing interest in focal therapy has led to the probing of various energy-based ablation techniques that selectively target cancerous lesions while sparing the surrounding normal prostate tissue [30].

Focal therapy represents a compromise between active surveillance and radical treatment, offering a minimally invasive option for patients with low- to intermediate-risk prostate cancer who seek oncologic control with fewer side effects [31,32,33]. Several focal therapy modalities have been developed, each employing different mechanisms to achieve tumor destruction while preserving surrounding healthy tissue. High-Intensity Focused Ultrasound (HIFU) utilizes ultrasound waves to generate thermal energy, inducing coagulative necrosis within the targeted lesion [34,35,36]. Cryotherapy, on the other hand, destroys cancerous cells by exposing them to extreme cold, leading to ice crystal formation and subsequent apoptosis [37,38,39]. Photodynamic Therapy (PDT) relies on the administration of photosensitizing agents that, when activated by specific wavelengths of light, produce cytotoxic reactive oxygen species that selectively target malignant cells [40,41]. Irreversible Electroporation (IRE) employs high-voltage electrical pulses to create permanent nanopores in cancer cell membranes, disrupting cellular homeostasis and triggering programmed cell death [42,43]. Among the laser-based techniques, Focal Laser Ablation (FLA) utilizes precisely controlled laser energy to generate localized thermal coagulation necrosis in tumor regions, effectively eradicating cancerous cells while minimizing damage to adjacent structures [44,45]. Among these modalities, FLA using the EchoLaser system with a transperineal approach to insert the laser applicator has emerged as a particularly promising approach due to its precision, minimal invasiveness, and ability to preserve prostate function. By employing ultrathin optical fibers to deliver diode laser energy with real-time ultrasound or fusion-guided imaging, EchoLaser technology enables precise tumor targeting, reducing the risk of overtreatment and preserving urinary and erectile function, making it an attractive alternative to traditional radical treatments [46,47,48].

The introduction of fiducial markers (FMs) has significantly improved the accuracy of image-guided prostate cancer treatments by providing reliable reference points for precise lesion targeting [49,50]. These radiopaque markers, implanted within the prostate gland, enhance visualization and positional tracking during both radiotherapy and focal ablation procedures, ensuring more accurate and reproducible treatment delivery [51]. One of their primary advantages is improved localization of the prostate gland, reducing treatment variability caused by anatomical shifts or patient movement [52,53]. Additionally, fiducial markers facilitate real-time tracking of organ motion, minimizing the risk of geographical miss, which is particularly crucial in highly targeted therapies [51,54]. By providing clear positional references, FMs allow for reduced treatment margins, leading to greater precision in dose delivery during radiotherapy or energy application in focal ablation. While fiducial markers have been extensively implemented in radiotherapy to enhance treatment accuracy, their role in focal ablation therapies, such as EchoLaser prostate cancer treatment, remains relatively understudied. The potential impact of fiducial markers on treatment precision, oncologic control, and procedural efficiency in focal therapy has not been comprehensively evaluated, highlighting the need for further clinical research to determine their efficacy and potential benefits in optimizing prostate cancer management.

Despite the growing adoption of EchoLaser technology in prostate cancer treatment, there is a lack of comparative studies evaluating the outcomes of EchoLaser focal therapy with and without fiducial marker assistance. Given the potential benefits of fiducials in enhancing precision and reducing recurrence rates, their role in focal ablation requires further investigation. This study aimed to bridge this knowledge gap by conducting a retrospective comparative analysis of EchoLaser therapy with and without fiducial markers in localized prostate cancer. By assessing oncologic outcomes, treatment-related complications, and procedural efficiency, we aimed to determine whether fiducial marker use provides a significant clinical advantage over non-assisted approaches. The results of this study could help optimize treatment protocols and refine the clinical application of EchoLaser therapy in PCa management.

## 2. Materials and Methods

### 2.1. Study Design

This study was conducted following the Strengthening the Reporting of Observational Studies in Epidemiology (STROBE) guidelines, ensuring methodological rigor, transparency in reporting, and reproducibility of results [55]. It was designed as a retrospective comparative cohort study, evaluating the efficacy, safety, and precision of EchoLaserfocal therapy for localized prostate cancer, with a particular focus on comparing outcomes between patients treated with and without fiducial markers (FMs).

This study was conducted at Athens Medical Center, Psychico Clinic, Greece, a referral center specializing in minimally invasive prostate cancer treatments. Patient recruitment took place between 18 September 2023, and 10 October 2024, with retrospective data collection continuing beyond this period. Ethical approval was granted by the Hospital’s Bioethics Committee (protocol number 559/24/01/2025), ensuring compliance with international ethical standards, including the Helsinki Declaration (1964 and subsequent amendments). All patients provided written informed consent prior to inclusion in the study.

### 2.2. Study Population

The study included male patients diagnosed with localized prostate adenocarcinoma who underwent EchoLaser focal laser ablation (FLA). Participants were selected based on predefined inclusion and exclusion criteria to ensure homogeneity within the cohort and to minimize confounding variables.

### 2.3. Inclusion Criteria

Patients were eligible for the study if they met the following criteria:

- Histologically confirmed localized prostate adenocarcinoma, confirmed by transperineal targeted biopsy.
- Gleason score ≤ 8, with stratification based on the International Society of Urological Pathology (ISUP) grade grouping.
- TNM staging between T1c and T2cN0M0, indicating no evidence of extraprostatic extension or nodal/metastatic involvement.
- Absence of prior curative treatment, including radical prostatectomy, external beam radiation therapy (EBRT), and brachytherapy.
- Favorable life expectancy (≥10 years) based on Charlson Comorbidity Index (CCI) assessment.
- Ability to undergo multiparametric MRI (mpMRI) for lesion characterization and treatment planning.
- Solitary or multiple localized lesions within the prostate gland.

### 2.4. Exclusion Criteria

The following exclusion criteria were applied:

- Contraindications to MRI scanning, such as implanted metallic devices, pacemakers, or severe claustrophobia.
- Uncontrolled coagulopathy or active bleeding disorders, as these increase the risk of hemorrhage following transperineal procedures.
- Severe cardiovascular or pulmonary comorbidities, including advanced heart failure (NYHA Class III-IV) and severe chronic obstructive pulmonary disease (COPD), which may increase procedural risk.
- History of prior prostate interventions, including previous focal therapy and irreversible electroporation (IRE), which may alter prostate anatomy.
- Allergy to the ultrasound contrast agent Sonovue™, used for enhanced imaging visualization.

### 2.5. Study Groups and Design

Patients included in this study were divided into two distinct treatment cohorts based on the use of fiducial markers (FMs) for lesion targeting and guidance during EchoLaser ablation. The classification into these groups aimed to evaluate whether fiducial-assisted targeting enhances treatment precision and procedural efficiency compared to standard ultrasound- or MRI-fusion guidance alone.

The first group, referred to as the Fiducial-assisted EchoLaser therapy group (FM+), consisted of patients who underwent radiopaque gold fiducial marker implantation during their diagnostic transperineal biopsy procedure. The rationale behind fiducial placement was to improve the localization of the prostate lesion during treatment planning, enhance visualization during MRI-fusion navigation, and facilitate real-time tracking of the targeted lesion throughout the laser ablation procedure. The fiducial markers were introduced transperineally under ultrasound guidance (TRUS), with strategic placement either within or adjacent to the tumor site to ensure optimal stability and minimal migration. This method allows for greater targeting precision, reducing the risk of misalignment due to prostate motion, deformation, and patient movement. Given that fiducial markers remain radiopaque and clearly visible on both MRI and transrectal ultrasound (TRUS), they serve as stable reference points throughout the entire treatment planning and execution process.

The second group, known as the Non-fiducial EchoLaser therapy group (FM−), included patients who underwent standard EchoLaser focal therapy without fiducial marker placement. In these cases, lesion targeting was performed using real-time ultrasound (US) guidance and MRI-fusion technology, relying solely on anatomical landmarks and direct lesion visualization without the aid of permanent reference markers. The FM− group followed the same imaging and treatment protocol as the FM+ group but did not have additional localization assistance from fiducials. While ultrasound and MRI fusion imaging provide relatively high accuracy, they are susceptible to interfractional organ motion, patient positioning variations, and soft tissue shifts, potentially leading to minor targeting discrepancies that may influence treatment outcomes.

Prior to treatment, all patients in both groups underwent a standardized pre-treatment evaluation protocol, ensuring consistency in patient selection and lesion assessment. This protocol included multiparametric magnetic resonance imaging (mpMRI) of the prostate, transperineal targeted biopsy, and baseline serum prostate-specific antigen (PSA) measurement. The mpMRI assessment followed the Prostate Imaging Reporting and Data System (PI-RADS) classification. This imaging approach provided critical information on tumor size, location, proximity to critical structures (e.g., neurovascular bundles, urethra, and rectum), and extracapsular extension risk, allowing for optimal treatment planning.

By incorporating both fiducial-assisted and standard MRI-fusion guided approaches within the study design, this comparative analysis aimed to determine whether fiducial marker use offers a significant clinical advantage over conventional targeting techniques. This study sought to evaluate whether fiducial markers contribute to improved precision, reduced recurrence rates, enhanced procedural efficiency, and fewer adverse effects in patients undergoing EchoLaser focal therapy for localized prostate cancer.

### 2.6. EchoLaser Treatment Protocol

The EchoLaser system, (Elesta S.p.A, Calenzano, Italy), is a multichannel diode laser system designed for real-time ultrasound- or MRI-fusion guided ablation of prostate lesions. This system operates using thin-caliber (≤1 mm diameter) optical fibers (Fiber Optic for PLA, Elesta S.p.A., Calenzano, Italy) inserted transperineally through introducer needles (Introducer, Elesta S.p.A., Calenzano, Italy) to deliver controlled laser-induced thermal energy to the lesion, leading to irreversible coagulative necrosis while preserving the surrounding structures.

The EchoLaser Smart Interface (ESI) was used to optimize fiber placement, energy dosing, and ablation zone monitoring, ensuring precise energy delivery. The pull-back technique was utilized for bigger lesions, allowing gradual and controlled thermal ablation while preventing overtreatment.

- Laser output: up to 7 watts per fiber,
- Total energy per lesion: up to 1800 J,
- Ablation dimensions: lateral width up to 1.2 cm, transverse dimension up to 1.5 cm and posterior safety margin up to 0.3 cm.
- Software Biopsee

### 2.7. Perioperative Management

In our protocol, lesion localization was based on pre-procedural multiparametric MRI, which was fused with transrectal ultrasound (TRUS) imaging using a 12 MHz probe. This integration enabled precise needle placement by correlating MRI-identified targets with real-time ultrasound guidance. Although conventional TRUS may not clearly visualize all prostate tumors, the MRI-TRUS correlation within the device platform improved targeting accuracy without requiring external fusion software. High-frequency micro-ultrasound (microUS) has shown potential in improving lesion visualization. However, this technology is currently unavailable in our center and is not widely adopted in Greece. As such, multiparametric MRI remains the primary imaging modality for lesion detection and treatment planning in our practice. While ablating the entire peripheral zone or hemigland may reduce the need for imaging guidance or fiducial markers, such an approach diverges from the principles of focal therapy. Our aim is to treat only the index lesion, as defined by MRI and biopsy, to preserve as much healthy prostate tissue as possible and minimize treatment-related morbidity. Therefore, fiducial markers remain a valuable tool in facilitating targeted focal ablation.

All procedures were performed under sedation anesthesia, with patients positioned in the lithotomy position to allow optimal transperineal access. Intravenous sedation and analgesia were administered as follows:

- Propofol 1% at a continuous infusion rate of 3 mL/kg/h, supplemented with 25–30 mg bolus doses as needed.
- Remifentanil (8 µg/mL solution) administered at a rate of 2 µg/kg/h for analgesia.
- Paracetamol (1 g IV) for perioperative pain management.
- Diclofenac (75 mg IV) as an additional anti-inflammatory and analgesic agent.
- Ondansetron (8 mg IV) for prophylaxis against postoperative nausea and vomiting.
- Omeprazole (40 mg IV) for gastric protection.

In order to minimize the risk of postoperative infections, prophylactic antibiotic therapy with ciprofloxacin (500 mg orally, administered one hour before the procedure, followed by 600 mg daily for five days) was employed, in accordance with international recommendations for infection prevention following transperineal interventions. To minimize the risk of urinary retention, dexamethasone (8 mg IV) and tamsulosin (0.4 mg orally) were administered once, one hour before the procedure. A 16 Fr Foley catheter was inserted intraoperatively and removed after 3 h and continuous bladder irrigation (CBI) with cold saline was applied to ensure urethral cooling and minimize the risk of thermal injury. Patients were discharged on the same day with detailed post-procedure care instructions. All procedures were performed by the same experienced urologist (corresponding author) to ensure consistency in technique and minimize variability in treatment outcomes.

### 2.8. Post-Treatment Monitoring and Follow-Up

Patients were regularly followed at six-month intervals for assessment of oncologic control, functional outcomes, and treatment-related safety. PSA levels were measured at 6 months post-procedure. Imaging assessments included multiparametric MRI (mpMRI) at 6 months. Repeat biopsy was considered if imaging findings suggested persistent or recurrent disease. Upon review of the follow-up data, all detected recurrences were found to be out-of-field, supporting the precision of lesion targeting and the effectiveness of the ablation technique within the treated area.

### 2.9. Statistical Analysis

Statistical analysis was performed using IBM SPSS Statistics v26.0, with continuous variables analyzed using Student’s *t*-test or the Mann–Whitney U test, depending on data normality, while categorical variables were compared using the chi-square test or Fisher’s exact test. A *p*-value of <0.05 was considered statistically significant, ensuring the robustness of the comparative outcomes between the FM+ and FM− groups.

## 3. Results

This section presents the demographic and clinical characteristics of the study population, including patient age, Gleason grade distribution, procedural details, and follow-up MRI findings at six months post-treatment. The data provide a comprehensive overview of the baseline characteristics and initial oncologic outcomes following EchoLaserfocal therapy. Additionally, the distribution of patients between the fiducial-assisted (FM+) and non-fiducial (FM−) groups is outlined, allowing for a comparative assessment of treatment precision and effectiveness.

Table 1 provides an overview of the demographic and clinical parameters of the study cohort. The mean age of the patients undergoing EchoLaser therapy was 72.36 ± 9.53 years, indicating that the treatment was primarily administered in an elderly population, consistent with the typical age range for localized prostate cancer management.

Regarding tumor grading, the mean Gleason group score was 2.36 ± 1.06, corresponding to an overall predominance of low- to intermediate-grade prostate cancer (Grade Groups 1–3). Specifically, 44% of patients (n = 22) were classified as Gleason Group 2 (Gleason score 3 + 4 = 7), representing the most common histopathologic subtype. Lower-grade tumors (Gleason Group 1, Gleason score ≤6) accounted for 20% of cases (n = 10), while Gleason Group 3 (4 + 3 = 7) was observed in 20% (n = 10) of patients. High-grade tumors were less frequent, with Grade Group 4 (Gleason score 8) observed in 12% (n = 6) and Grade Group 5 (Gleason score 9–10) in only 4% (n = 2) of cases. Although focal therapy is typically indicated for patients with low- to intermediate-risk prostate cancer, two elderly patients (over 80 years old) with Grade Group 5 disease were included in this cohort. In these cases, radical prostatectomy was deemed unsuitable due to advanced age and comorbidities, and focal therapy was selected as a less invasive alternative to optimize tumor control while minimizing treatment-related morbidity. The median Charlson Comorbidity Index (CCI) score among the study cohort was three, indicating a moderate degree of comorbidity burden. The median lesion volume, as assessed by preoperative multiparametric MRI, was 4.1 cm^3^.

At the six-month follow-up MRI assessment, 80% of patients (n = 40) exhibited no evidence of residual tumor, indicating a high initial treatment success rate. However, 20% (n = 10) showed persistent or recurrent tumor activity, necessitating further evaluation or potential additional intervention. The presence of residual disease on imaging highlights the need for ongoing surveillance and possibly adjunctive treatment in select cases. In terms of fiducial marker use, 62% of patients (n = 31) underwent fiducial-assisted targeting (FM+), whereas 38% (n = 19) were treated without fiducials (FM−). The mean operative time for EchoLaser therapy was 38.16 ± 6.56 min, reflecting the minimally invasive and efficient nature of the procedure. Throughout the six-month follow-up period, no cases of perineal pain, perineal hematoma, hematuria, hematospermia, erectile dysfunction, or urinary incontinence were observed among the treated patients, indicating a favorable safety and tolerability profile for the EchoLaser focal therapy approach.

**Figure 1:**
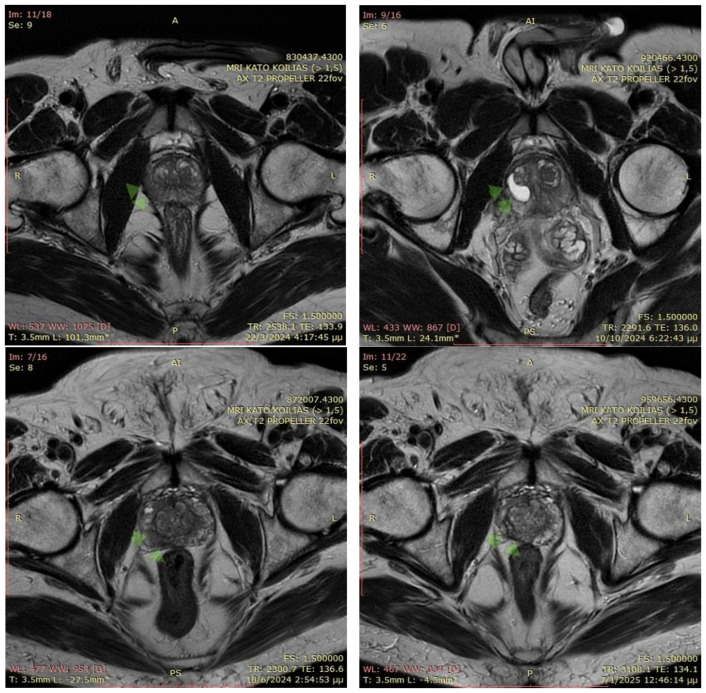
MRI of two patients pre-biopsy and after the EchoLaser therapy with fiducial markers procedure. The arrows in the figure indicate the locations of the lesions.

The MRI images illustrate axial T2-weighted scans of the prostate before and after EchoLaser focal therapy for prostate cancer with fiducial markers. In the pre-treatment MRI (22 March 2024 and 18 June 2024, respectively), the prostate appears relatively homogeneous, with a marked region of interest that may indicate a cancerous lesion. Fiducial markers were placed prior to the procedure to enhance precision in targeting the tumor. In the post-treatment MRI (10 October 2024 and 7 January 2025, respectively), significant structural alterations are visible within the prostate, including a well-defined hypointense area corresponding to necrotic tissue, a typical post-ablation finding. The presence of fiducial markers ensures accurate monitoring of treatment effects, confirming that the laser ablation successfully induced thermal damage to the targeted region while preserving surrounding healthy tissue (Figure 1).

Table 2 presents the changes in serum prostate-specific antigen (PSA) levels before biopsy and at six months post-treatment, reflecting the biochemical response to EchoLaserfocal therapy in patients with localized prostate cancer. The mean pre-biopsy PSA level was 10.26 ± 14.99 ng/mL, with a median value of 5.95 ng/mL (IQR: 4.34–9.06 ng/mL), indicating a broad distribution of baseline PSA values across the cohort. The wide standard deviation and the presence of a maximum pre-biopsy PSA level of 100.00 ng/mL suggest that a subset of patients exhibited markedly elevated PSA values, potentially indicative of higher tumor burden, larger lesion volume, or increased inflammatory activity within the prostate.

At six months post-procedure, a significant reduction in PSA levels was observed, with a mean post-treatment PSA of 2.70 ± 2.67 ng/mL and a median value of 1.90 ng/mL (IQR: 1.11–3.26 ng/mL). This 74% median PSA decline reflects successful tumor ablation and a reduction in overall prostatic tumor activity, consistent with the expected biochemical response following effective focal therapy. Notably, the minimum PSA value recorded post-treatment was 0.00 ng/mL, which may indicate complete tumor eradication or a profound treatment response in select cases. However, the maximum post-treatment PSA level remained elevated at 15.00 ng/mL, suggesting that a subset of patients exhibited biochemical persistence or early recurrence, which aligns with the 20% rate of residual tumor observed on six-month follow-up MRI scans (Table 1).

The observed PSA decline at six months was clinically significant, as a post-treatment PSA reduction of ≥50% is generally considered a positive prognostic indicator in focal therapy outcomes. However, the variability in PSA response warrants further stratification based on the Gleason score, lesion characteristics, and fiducial marker use to determine whether specific patient subgroups derive greater oncologic benefit from EchoLaser treatment. Finally, the pattern of PSA reduction was consistent across both groups, suggesting that the presence of fiducial markers does not significantly alter the PSA trajectory at six months post-treatment and this is the reason for the standard deviation being higher than the mean value.

In the non-fiducial EchoLaser therapy group (FM−), one case of post-treatment complication (urinary retention occurring three months post-procedure) was reported, highlighting potential limitations in lesion targeting and procedural precision when fiducial markers were not used. Although urinary retention is a known adverse effect of focal therapy, its occurrence in the non-fiducial group raises concerns regarding peri-prostatic tissue involvement, possibly due to minor inaccuracies in lesion targeting.

Table 3 presents a comparative analysis of key demographic and procedural variables between the two treatment groups, stratified by the use of fiducial markers (FM+ vs. FM−). Statistical significance was determined using Student’s *t*-test for normally distributed variables and the Mann–Whitney U test for non-normally distributed data, with all reported *p*-values being <0.01, indicating highly significant differences. The mean age of patients in the FM− group was significantly higher (78.37 ± 8.68 years) than that of the patients in the FM+ group (68.68 ± 8.14 years), with a t-value of 3.985 (*p* < 0.01). A significant difference was observed in tumor aggressiveness between the two groups, with the mean Gleason Group score being 3.00 ± 1.25 in the FM− group compared to 1.97 ± 0.71 in the FM+ group (U = 146.500, *p* < 0.01). This indicates that higher-grade tumors (more aggressive disease) were more prevalent in the non-fiducial group, while lower-grade tumors (Gleason ≤ 3 + 4) were more frequently treated with fiducial-assisted targeting. The duration of the procedure was significantly longer in the FM− group (45.79 ± 2.92 min) compared to the FM+ group (33.48 ± 2.41 min), with a U-value of 0.000 (*p* < 0.01).

Table 4 presents a crosstabulation analysis examining the relationship between fiducial marker use and MRI-confirmed oncologic outcomes at six months post-EchoLaser focal therapy. A chi-square test (χ^2^ = 5.433, *p* < 0.05) indicated a statistically significant association between fiducial marker use and post-treatment imaging results, suggesting that fiducial-assisted targeting may contribute to improved lesion eradication rates.

Table 5 presents a Spearman correlation analysis examining the association between the duration of EchoLaser focal therapy and key clinical variables, including patient age and Gleason grade. Spearman’s rho (ρ) was used to assess non-parametric correlations, with statistical significance set at *p* < 0.05 and *p* < 0.01 thresholds for moderate and strong correlations, respectively. A positive correlation was observed between age and duration of operation (ρ = 0.44, *p* < 0.01), indicating that older patients required longer procedural times. Similarly, a moderate positive correlation was found between Gleason grade and operative duration (ρ = 0.40, *p* < 0.01), suggesting that higher-grade tumors were associated with longer procedures.

## 4. Discussion

The findings of this study suggest that EchoLaser focal therapy is an effective and minimally invasive treatment for localized prostate cancer, particularly in low- to intermediate-risk cases. The six-month follow-up MRI results demonstrate an 80% rate of complete tumor ablation, while PSA levels exhibited a 74% median reduction, reflecting successful oncologic control. Additionally, the use of fiducial markers (FM+) was associated with better treatment precision and reduced recurrence rates. It is important to note that non-homogeneous groups (the FM+ group and the FM− group) in terms of baseline characteristics (in particular Gleason Score) could have affected the differences in recurrence rate.

One of the findings was the significant difference in procedure duration between the FM+ and FM− groups. Patients treated with fiducial-assisted targeting had shorter operative times (33.48 ± 2.41 min) compared to those without fiducials (45.79 ± 2.92 min, *p* < 0.01). This suggests that fiducial markers improve lesion localization efficiency, reducing the need for extensive intraoperative imaging adjustments and reorientation.

From a safety perspective, the overall complication rate was low, with only case event of urinary retention (3 months post-procedure) observed exclusively in the FM− group. This finding could highlight potential limitations in lesion coverage and peri-prostatic tissue involvement when fiducial markers are not used.

The results of this study align with the existing literature on transperineal laser ablation (TPLA) for focal prostate cancer therapy, particularly the studies by Manenti et al. (2024) [45] and Iacovelli et al. (2024) [44]. Manenti et al. conducted an interventional pilot study assessing the three-year oncologic and functional outcomes of TPLA in low- to intermediate-risk prostate cancer. Their findings demonstrated an 80% MRI-confirmed tumor eradication rate, with a 60% mean PSA reduction and no evidence of malignant disease on biopsies at 36 months in 50% of cases [45]. Similarly, Iacovelli et al. reported an 87.5% success rate at 12 months, with no significant deterioration in urinary continence or erectile function [44].

In comparison, the 80% tumor eradication rate observed in the present study closely mirrors the findings of Manenti et al., suggesting that EchoLaser therapy achieves similar oncologic outcomes to other focal laser ablation techniques. However, the PSA reduction in this study was higher (74%) than the 60% reported by Manenti et al., potentially reflecting differences in patient selection, laser energy parameters, or post-treatment follow-up duration. Additionally, the 12.5% recurrence rate reported by Iacovelli et al. at 12 months is comparable to the 20% MRI-confirmed recurrence rate in the present study at six months, though longer-term follow-up is required to establish definitive recurrence rates.

Moreover, the data suggest that fiducial marker use significantly improves procedural accuracy and reduces operative time, underscoring the potential clinical benefits of integrating fiducial guidance into routine focal therapy protocols. Given that FLA studies have not systematically incorporated fiducial markers, future comparative trials should investigate whether fiducial-assisted laser therapy yields superior oncologic outcomes compared to standard MRI-ultrasound fusion guidance alone.

Despite its strengths, this study has several limitations. First of all, this is a retrospective study. Second, the follow-up period is currently limited to six months, which precludes long-term assessment of oncologic durability and biochemical recurrence-free survival (bRFS). Future studies should include longer follow-up intervals (12, 24, and 36 months) with repeat MRI and biopsy assessments to determine whether the early oncologic advantages of fiducial-assisted targeting translate into durable disease control.

Third, functional outcomes such as urinary and sexual health were not assessed, limiting the ability to compare quality-of-life metrics with those reported in the literature. Fourth, while the statistically significant association between fiducial use and improved oncologic outcomes suggests a potential causal relationship, additional prospective, randomized controlled trials (RCTs) are needed to confirm these findings. A direct head-to-head comparison between fiducial-assisted EchoLaser therapy and non-fiducial FLA would provide valuable insights into the true impact of fiducial markers on focal therapy outcomes. Given the novelty of EchoLaser focal therapy and the limited number of comparative studies available in the current literature, our findings should be interpreted as preliminary. Further large-scale studies and longer-term follow-up are necessary to confirm the oncologic efficacy, safety profile, and functional outcomes observed in this initial experience.

## 5. Conclusions

Fiducial marker placement can be helpful for TPLA procedures and can be considered an alternative to more time-consuming and expensive fusion technology, which is less accurate for targeting.

## Figures and Tables

**Table 1:** Baseline Demographic, Clinical, and Procedural Characteristics of the Study Population.

	Frequency	Percentage
Age (in years) *	72.36 ± 9.53	
Gleason Groups *	2.36 ± 1.06	
Group 1	10	20.00
Group 2	22	44.00
Group 3	10	20.00
Group 4	6	12.00
Group 5	2	4.00
Fiducial		
No	19	38.00
Yes	31	62.00
Duration of operation (in minutes) *	38.16 ± 6.56	
MRI (after 6 months)		
Negative	40	80.00
Positive	10	20.00

Notes: * Mean ± Standard Deviation; Grade group 1: Gleason score 6 or lower (low-grade cancer); Grade group 2: Gleason score 3 + 4 = 7 (medium-grade cancer); Grade group 3: Gleason score 4 + 3 = 7 (medium-grade cancer); Grade group 4: Gleason score 8 (high-grade cancer); Grade group 5: Gleason score 9 to 10 (high-grade cancer).

**Table 2:** Serum PSA Following EchoLaser Focal Therapy.

	PSA	
	Pre Biopsy	After 6 Months
Mean ± Standard Deviation	10.26 ± 14.99	2.70 ± 2.67
Median (IQR)	5.95 (4.34–9.06)	1.90 (1.11–3.26)
Minimum	2.38	0.00
Maximum	100.00	15.00

**Table 3:** Comparative Analysis of Patient and Procedural Characteristics Between Fiducial-Assisted (FM+) and Non-Fiducial (FM−) Groups.

	Fiducial		t(48)/U	*p*
	No	Yes		
	M ± SD	M ± SD		
Age ^a^	78.37 ± 8.68	68.68 ± 8.14	3.985	<0.01
Gleason Groups ^b^	3.00 ± 1.25	1.97 ± 0.71	146.500	<0.01
Duration of operation (in minutes) ^b^	45.79 ± 2.92	33.48 ± 2.41	0.000	<0.01

Notes: ^a^ Student’s *t*-test. ^b^ Mann–Whitney. M = Mean, SD = Standard Deviation.

**Table 4:** Impact of Fiducial Markers on Six-Month MRI Outcomes Following EchoLaser Focal Therapy.

			Fiducial		Total	χ^2^
			No	Yes		
MRI (after 6 months)	Negative	Count	12	28	40	χ^2^ = 5.433 *p* < 0.05
		% within MRI (after 6 months)	30.0%	70.0%	100.0%	
	Positive	Count	7	3	10	
		% within MRI (after 6 months)	70.0%	30.0%	100.0%	
Total		Count	19	31	50	
		% within MRI (after 6 months)	38.0%	62.0%	100.0%	

**Table 5:** Correlation Between Operative Duration and Clinical Variables in EchoLaser Focal Therapy.

		Duration of Operation (in Minutes)
Age	Correlation Coefficient	0.44 *
	Sig. (2-tailed)	<0.01
	N	50
Gleason Groups	Correlation Coefficient	0.40 *
	Sig. (2-tailed)	<0.01
	N	50

Notes: * Correlation is significant at the 0.01 level (2-tailed).

## Data Availability

The data can be shared up on request.

## References

[1] Zhang Y., Wang P., Jia Z., Zheng Z., Wang J., Liang H. (2025). Global burden and risk factors of male cancers from 1990 to 2021, with forecasts to 2040. Sci. Rep..

[2] Bergengren O., Pekala K.R., Matsoukas K., Fainberg J., Mungovan S.F., Bratt O., Bray F., Brawley O., Luckenbaugh A.N., Mucci L. (2023). 2022 Update on Prostate Cancer Epidemiology and Risk Factors—A Systematic Review. Eur. Urol..

[3] Berenguer C.V., Pereira F., Câmara J.S., Pereira J.A.M. (2023). Underlying Features of Prostate Cancer—Statistics, Risk Factors, and Emerging Methods for Its Diagnosis. Curr. Oncol..

[4] Wong M.C.S., Goggins W.B., Wang H.H.X., Fung F.D.H., Leung C., Wong S.Y.-S., Ng C.-F., Sung J.J.Y. (2016). Global Incidence and Mortality for Prostate Cancer: Analysis of Temporal Patterns and Trends in 36 Countries. Eur. Urol..

[5] Wang L., Lu B., He M., Wang Y., Wang Z., Du L. (2022). Prostate Cancer Incidence and Mortality: Global Status and Temporal Trends in 89 Countries From 2000 to 2019. Front. Public Health.

[6] Serdà-Ferrer B.C., Sanvisens A., Fuentes-Raspall R., Puigdemont M., Farré X., Vidal-Vila A., Rispau-Pagès M., Baltasar-Bagué A., Marcos-Gragera R. (2023). Significantly reduced incidence and improved survival from prostate cancer over 25 years. BMC Public Health.

[7] Chung J.S., Morgan T.M., Hong S.K. (2020). Clinical implications of genomic evaluations for prostate cancer risk stratification, screening, and treatment: A narrative review. Prostate Int..

[8] Olah C., Mairinger F., Wessolly M., Joniau S., Spahn M., Julio M.K.-D., Hadaschik B., Soós A., Nyirády P., Győrffy B. (2024). Enhancing risk stratification models in localized prostate cancer by novel validated tissue biomarkers. Prostate Cancer Prostatic Dis..

[9] Pedrani M., Barizzi J., Salfi G., Nepote A., Testi I., Merler S., Castelo-Branco L., Mestre R.P., Turco F., Tortola L. (2025). The Emerging Predictive and Prognostic Role of Aggressive-Variant-Associated Tumor Suppressor Genes Across Prostate Cancer Stages. Int. J. Mol. Sci..

[10] Ciccarese C., Massari F., Iacovelli R., Fiorentino M., Montironi R., Di Nunno V., Giunchi F., Brunelli M., Tortora G. (2017). Prostate cancer heterogeneity: Discovering novel molecular targets for therapy. Cancer Treat. Rev..

[11] Ongün Ş., Sarıkaya A.E., Yılmaz S.H.B., Sevgi B., Çelik S., Şen V., Tuna B., Yörükoğlu K., Aslan G., Mungan M.U. (2024). Long-term Surveillance Outcomes of Prostate Cancer Patients Eligible for Active Surveillance but Who Underwent Radical Prostatectomy. J. Urol. Surg..

[12] Eggener S.E., Berlin A., Vickers A.J., Paner G.P., Wolinsky H., Cooperberg M.R. (2022). Low-Grade Prostate Cancer: Time to Stop Calling It Cancer. J. Clin. Oncol..

[13] Baboudjian M., Breda A., Rajwa P., Gallioli A., Gondran-Tellier B., Sanguedolce F., Verri P., Diana P., Territo A., Bastide C. (2022). Active Surveillance for Intermediate-risk Prostate Cancer: A Systematic Review, Meta-analysis, and Metaregression. Eur. Urol. Oncol..

[14] An J.Y., Sidana A., Choyke P.L., Wood B.J., Pinto P.A., Türkbey İ.B. (2017). Multiparametric Magnetic Resonance Imaging for Active Surveillance of Prostate Cancer. Balk. Med. J..

[15] Chappidi M.R., Lin D.W., Westphalen A.C. (2025). Role of MRI in Active Surveillance of Prostate Cancer. Semin. Ultrasound CT MRI.

[16] Press B.H., Jones T., Olawoyin O., Lokeshwar S.D., Rahman S.N., Khajir G., Lin D.W., Cooperberg M.R., Loeb S., Darst B.F. (2022). Association Between a 22-feature Genomic Classifier and Biopsy Gleason Upgrade During Active Surveillance for Prostate Cancer. Eur. Urol. Open Sci..

[17] Komisarenko M., Wong L.-M., Richard P.O., Timilshina N., Toi A., Evans A., Zlotta A., Kulkarni G., Hamilton R., Fleshner N. (2016). An Increase in Gleason 6 Tumor Volume While on Active Surveillance Portends a Greater Risk of Grade Reclassification with Further Followup. J. Urol..

[18] Hagmann S., Ramakrishnan V., Tamalunas A., Hofmann M., Vandenhirtz M., Vollmer S., Hug J., Niggli P., Nocito A., Kubik-Huch R.A. (2022). Two Decades of Active Surveillance for Prostate Cancer in a Single-Center Cohort: Favorable Outcomes after Transurethral Resection of the Prostate. Cancers.

[19] Boladeras A., Martinez E., Ferrer F., Gutierrez C., Villa S., Pera J., Guedea F. (2016). Localized prostate cancer treated with external beam radiation therapy: Long-term outcomes at a European comprehensive cancer centre. Rep. Pract. Oncol. Radiother..

[20] Chen L., Li Q., Wang Y., Zhang Y., Ma X. (2017). Comparison on efficacy of radical prostatectomy versus external beam radiotherapy for the treatment of localized prostate cancer. Oncotarget.

[21] Numakura K., Kobayashi M., Muto Y., Sato H., Sekine Y., Sobu R., Aoyama Y., Takahashi Y., Okada S., Sasagawa H. (2023). The Current Trend of Radiation Therapy for Patients with Localized Prostate Cancer. Curr. Oncol..

[22] Boorjian S.A., Karnes R.J., Viterbo R., Rangel L.J., Bergstralh E.J., Horwitz E.M., Blute M.L., Buyyounouski M.K. (2011). Long-term survival after radical prostatectomy versus external-beam radiotherapy for patients with high-risk prostate cancer. Cancer.

[23] Stanford J.L., Feng Z., Hamilton A.S., Gilliland F.D., Stephenson R.A., Eley J.W., Albertsen P.C., Harlan L.C., Potosky A.L. (2000). Urinary and Sexual Function After Radical Prostatectomy for Clinically Localized Prostate Cancer: The Prostate Cancer Outcomes Study. JAMA.

[24] Rossi F., Marino F., Gandi C., Bizzarri F.P., Campetella M., Bientinesi R., Silvaggi M., Sacco E. (2025). Relationship Between Post-Prostatectomy urinary Incontinence, Sexual Functions, and Dyadic Adjustment: A Cross-Sectional Study. Urologia.

[25] Haglind E., Carlsson S., Stranne J., Wallerstedt A., Wilderäng U., Thorsteinsdottir T., Lagerkvist M., Damber J.-E., Bjartell A., Hugosson J. (2015). Urinary Incontinence and Erectile Dysfunction After Robotic Versus Open Radical Prostatectomy: A Prospective, Controlled, Nonrandomised Trial. Eur. Urol..

[26] Gacci M., De Nunzio C., Sakalis V., Rieken M., Cornu J.N., Gravas S. (2023). Latest Evidence on Post-Prostatectomy Urinary Incontinence. J. Clin. Med..

[27] Li G., Li Y., Wang J., Gao X., Zhong Q., He L., Li C., Liu M., Liu Y., Ma M. (2021). Guidelines for radiotherapy of prostate cancer (2020 edition). Precis. Radiat. Oncol..

[28] Suriano F., Altobelli E., Sergi F., Buscarini M. (2013). Bladder Cancer After Radiotherapy for Prostate Cancer. Rev. Urol..

[29] Jahreiß M.C., Heemsbergen W.D., Aben K.K.H., Incrocci L. (2024). Risk factors for secondary bladder cancer following prostate cancer radiotherapy. Transl. Androl. Urol..

[30] Valerio M., Ahmed H.U., Emberton M., Lawrentschuk N., Lazzeri M., Montironi R., Nguyen P.L., Trachtenberg J., Polascik T.J. (2014). The Role of Focal Therapy in the Management of Localised Prostate Cancer: A Systematic Review. Eur. Urol..

[31] Eggener S., Salomon G., Scardino P.T., De la Rosette J., Polascik T.J., Brewster S. (2010). Focal Therapy for Prostate Cancer: Possibilities and Limitations. Eur. Urol..

[32] Klotz L. (2015). Active surveillance and focal therapy for low-intermediate risk prostate cancer. Transl. Androl. Urol..

[33] Nomura T., Mimata H. (2012). Focal Therapy in the Management of Prostate Cancer: An Emerging Approach for Localized Prostate Cancer. Adv. Urol..

[34] Napoli A., Alfieri G., Scipione R., Leonardi A., Fierro D., Panebianco V., De Nunzio C., Leonardo C., Catalano C. (2020). High-intensity focused ultrasound for prostate cancer. Expert Rev. Med. Devices.

[35] Elhelf I.A.S., Albahar H., Shah U., Oto A., Cressman E., Almekkawy M. (2018). High intensity focused ultrasound: The fundamentals, clinical applications and research trends. Diagn. Interv. Imaging.

[36] Izadifar Z., Izadifar Z., Chapman D., Babyn P. (2020). An Introduction to High Intensity Focused Ultrasound: Systematic Review on Principles, Devices, and Clinical Applications. J. Clin. Med..

[37] Aker M.N., Brisbane W.G., Kwan L., Gonzalez S., Priester A.M., Kinnaird A., Delfin M.K., Felker E., Sisk A.E., Kuppermann D. (2023). Cryotherapy for partial gland ablation of prostate cancer: Oncologic and safety outcomes. Cancer Med..

[38] Kotamarti S., Polascik T.J. (2022). Focal cryotherapy for prostate cancer: A contemporary literature review. Ann. Transl. Med..

[39] Baust J.G., Gage A.A., Bjerklund Johansen T.E., Baust J.M. (2013). Mechanisms of Cryoablation: Clinical Consequences on Malignant Tumors. Cryobiology.

[40] Mesquita M.Q., Ferreira A.R., Neves Mda G.P.M.S., Ribeiro D., Fardilha M., Faustino M.A.F. (2021). Photodynamic therapy of prostate cancer using porphyrinic formulations. J. Photochem. Photobiol. B..

[41] Gheewala T., Skwor T., Munirathinam G. (2017). Photosensitizers in prostate cancer therapy. Oncotarget.

[42] Geboers B., Scheltema M.J., Jung J., Bakker J., Timmer F.E., Cerutti X., Katelaris A., Doan P., Gondoputro W., Blazevski A. (2025). Irreversible electroporation of localised prostate cancer downregulates immune suppression and induces systemic anti-tumour T-cell activation—IRE-IMMUNO study. BJU Int..

[43] Faiella E., Santucci D., Vertulli D., Vergantino E., Vaccarino F., Perillo G., Zobel B.B., Grasso R.F. (2024). Irreversible Electroporation (IRE) for Prostate Cancer (PCa) Treatment: The State of the Art. J. Pers. Med..

[44] Iacovelli V., Carilli M., Bertolo R., Forte V., Vittori M., Filippi B., Di Giovanni G., Cipriani C., Petta F., Maiorino F. (2024). Transperineal Laser Ablation for Focal Therapy of Localized Prostate Cancer: 12-Month Follow-up Outcomes from a Single Prospective Cohort Study. Cancers.

[45] Manenti G., Perretta T., Nezzo M., Fraioli F.R., Carreri B., Gigliotti P.E., Micillo A., Malizia A., Di Giovanni D., Ryan C.P. (2024). Transperineal Laser Ablation (TPLA) Treatment of Focal Low–Intermediate Risk Prostate Cancer. Cancers.

[46] Cocci A., Pezzoli M., Bianco F., Blefari F., Bove P., Cornud F., De Rienzo G., Destefanis P., Di Trapani D., Giacobbe A. (2023). Transperineal laser ablation of the prostate as a treatment for benign prostatic hyperplasia and prostate cancer: The results of a Delphi consensus project. Asian J. Urol..

[47] Sessa F., Polverino P., Bisegna C., Siena G., Re M.L., Spatafora P., Pecoraro A., Rivetti A., Conte F.L., Cocci A. (2022). Transperineal laser ablation of the prostate with EchoLaser^TM^ system: Perioperative and short-term functional and sexual outcomes. Front. Urol..

[48] Tzelves L., Nagasubramanian S., Pinitas A., Juliebø-Jones P., Madaan S., Sienna G., Somani B. (2023). Transperineal laser ablation as a new minimally invasive surgical therapy for benign prostatic hyperplasia: A systematic review of existing literature. Ther. Adv. Urol..

[49] Osman S.O.S., Russell E., King R.B., Crowther K., Jain S., McGrath C., Hounsell A.R., Prise K.M., McGarry C.K. (2019). Fiducial markers visibility and artefacts in prostate cancer radiotherapy multi-modality imaging. Radiat. Oncol..

[50] Brown K., Ghita M., Prise K.M., Butterworth K.T. (2023). Feasibility and guidelines for the use of an injectable fiducial marker (BioXmark^®^) to improve target delineation in preclinical radiotherapy studies using mouse models. F1000Research.

[51] Mahdavi A., Mofid B., Taghizadeh-Hesary F. (2023). Intra-prostatic gold fiducial marker insertion for image-guided radiotherapy (IGRT): Five-year experience on 795 patients. BMC Med. Imaging.

[52] Mirzaei M., Gill S., Sabet M., Ebert M.A., Rowshanfarzad P., Kendrick J., Jacques A., Herbert C., Croker J., Bydder S. (2025). Treatment efficiency and quality improvement via double imaging modality (DIM) versus single imaging modality (SIM) image-guided radiotherapy for prostate cancer. Tech. Innov. Patient Support Radiat. Oncol..

[53] Gurjar O.P., Arya R., Goyal H. (2020). A study on prostate movement and dosimetric variation because of bladder and rectum volumes changes during the course of image-guided radiotherapy in prostate cancer. Prostate Int..

[54] O’neill A.G.M., Jain S., Hounsell A.R., O’sullivan J.M. (2016). Fiducial marker guided prostate radiotherapy: A review. Br. J. Radiol..

[55] Elm Evon Altman D.G., Egger M., Pocock S.J., Gøtzsche P.C., Vandenbroucke J.P. (2007). Strengthening the reporting of observational studies in epidemiology (STROBE) statement: Guidelines for reporting observational studies. BMJ Br. Med. J..

